# Supplementary material for: General Method to Increase Carboxylic Acid Content on Nanodiamonds
Source: Molecules. 2022 Jan 23;27(3):736. doi: 10.3390/molecules27030736 (PMC8838522; doi:10.3390/molecules27030736)
Supplement: Supplementary file 1 [file molecules-27-00736-s001.zip › Molecules_1496189_SI_revision_2_final.pdf]

Supporting Information
General Method to Increase Carboxylic Acid Content on Nanodiamonds

Ganesh Shenoy[†*], Jessica Ettedgui[‡*], Chandrasekhar Mushti[‡], Jennifer Hong[†], Kelly Lane[‡], Burchelle Blackman[‡], Hak-Sung Jung[†], Yasuharu Takagi[†], Yeonee Seol[†], Martin Brechbiel[§], Rolf E. Swenson[‡], and Keir C. Neuman[†]

[†]Laboratory of Single Molecule Biophysics, National Heart Lung and Blood Institute, National Institutes of Health, Building 50 Room 2134, Bethesda, MD 20892.
[‡]Chemistry and Synthesis Center, National Heart Lung and Blood Institute, National Institutes of Health, Building B Room 3040, 9800 Medical Center Drive, Rockville, MD 20850.
[§]Radiation Oncology Branch, National Cancer Institute, National Institutes of Health, Building 10, Room B3B69, Bethesda, MD 20892.

1. **Thermogravimetric analysis**
    1.1. FNDs TGA starting material
    1.2. TGA and DTG of FNDs-PEG$_6$-Biotin
    1.3. Biotin Calculations

2. **Quantifying biotin on nanodiamond surface.**

3. **Evanescent wave penetration depth measurement and calibration.**

4. **ND-biotin optical trapping**.

5. **Synthetic Methodology**
    5.1. General method for Carboxylic acid enrichment of oxidized FNDs
        5.1.1. Coupling carboxylic acid enriched FNDs to amine-PEG$_6$-azide
        5.1.2. Coupling carboxylic acid enriched FNDs to amine-PEG$_{2000}$-biotin
    5.2. General method for Carboxylic acid enrichment of reduced FNDs
        5.2.1. Carboxylic acid enrichment of FNDs
        5.2.2. Synthesis of acyl chloride FNDs
        5.2.3. Coupling of carboxylic acid enriched FNDs with tags:
            a) FND-amido-PEG$_6$-azide
            b) FND-amido- PEG$_6$-Propargyl
            c) FND-amido-PEG$_6$-Biotin
            d) FND-amido-ethyl-PEG$_4$-Maleimide
            e) FND-amido-PEG$_3$-amido-t-Boc-N
            f) FND-amidoethyl-PEG$_4$-carboxylic acid
            g) FND-amidoethyl-PEG$_4$-acetyl chloride
            h) FND-amido-PEG$_4$-DBCO
            i) FND-amido-Halo Tag
            j) FND-amido-SNAP Tag
            k) FND-amido-Clip tag

Thermogravimetric Analysis

TGA and DTG curves provide the decomposition rate and evaluate mass-loss steps by normalizing weight loss rate to the heating rate. TGA measurements were performed with ~10 mg of material in a platinum pan using a Q50 thermogravimetric analyzer (TA Instruments, USA). All measurements were performed in an Ar atmosphere (Airgas, USA, argon, compressed, 2.2, UN1066) after 2 h of flushing the setup with Ar at room temperature to ensure complete removal of air. The weight loss was recorded over the following temperature program:

Isothermal at 25°C for 5 minutes
Heat 25 to 120°C at 10°C/min
Isothermal at 120°C for 10 minutes to ensure complete removal of residual volatile solvents
Heat 120 to 1200 °C at 10 °C/min

The temperature-dependent mass differential was calculated from ~10,000 data points. The first derivative of the mass with respect to the temperature (DTG) was first obtained; then, zero crossings of the second derivative of the mass with respect to the temperature were used to establish the mass loss stages. Data processing was done using MATLAB (The Mathworks, Natick, MA).

1.1. TGA analysis of bare FNDs HPHT

A

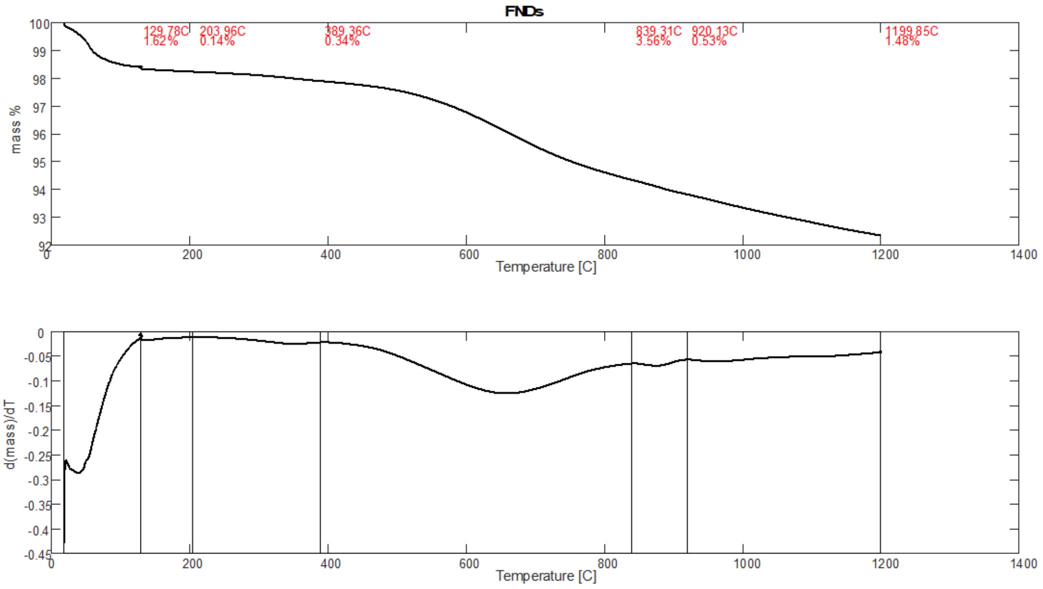

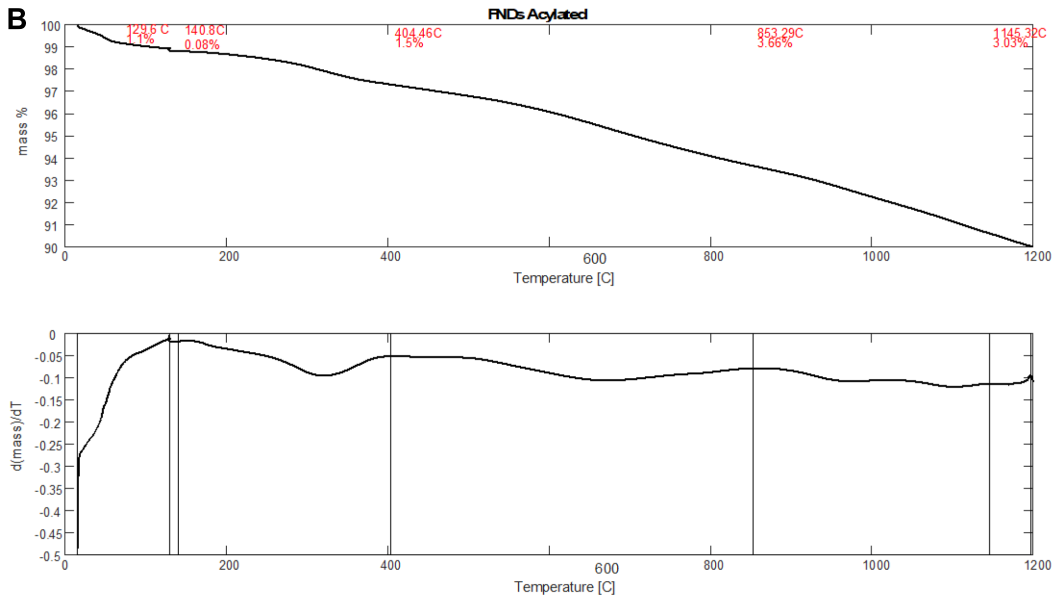

Figure S1: TGA analysis of FNDs. TGA (top) and DTG (bottom) curves of reduced FNDs starting material (A) and FNDs after acylation (B).

Accounting for the NDs contribution in the biotinylated FNDs:

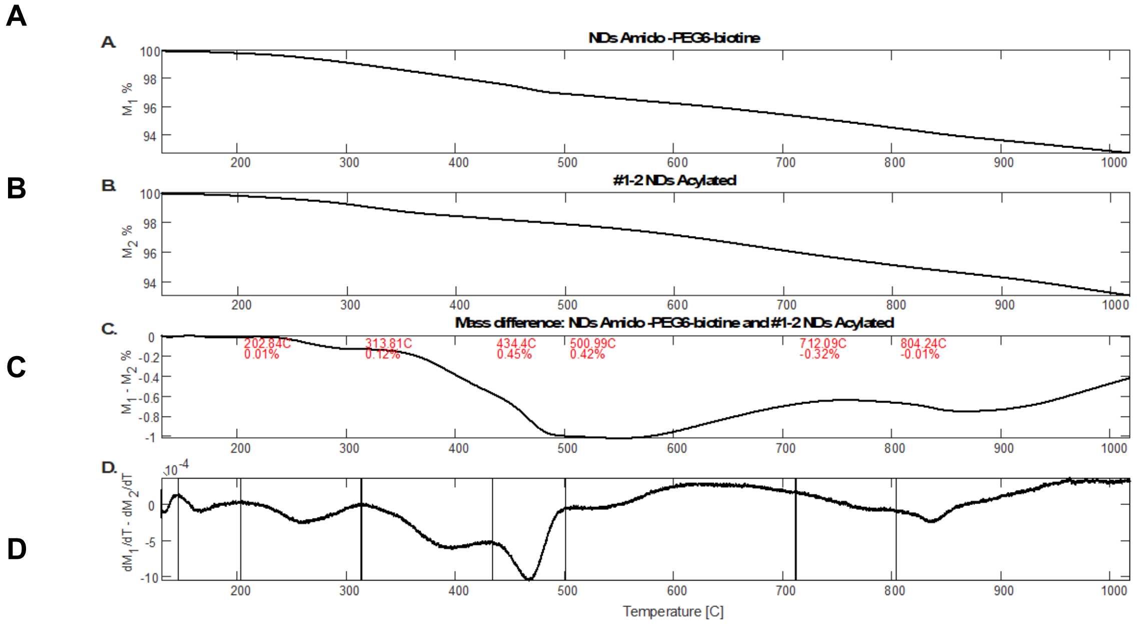

Figure S2: (A) TGA of amido-PEG$_6$-biotin functionalized FNDs. (B) TGA of acylated FNDs. (C) Mass difference between amido-PEG$_6$- biotin functionalized FNDs and acylated FNDs. (D) DTG of the TGA difference. Only, material added to the surface of the FNDs is measured through this process.

Approximately, 0.42 % of the nanodiamond mass corresponds to the biotin labeling.

1.2. Biotin Surface Density Calculations

For biotinylated PEG_6 FNDs 0.42% of the FND mass corresponds to biotinylated PEG_6 with mass M= 548.7 g/mol (https://broadpharm.com/web/product.php?catalog=BP-22116)
For 1 mg of sample there are 0.0042 mg of biotin PEG_6 with M= 548.7 g/mol
So, 7.654×10^{-9} mol of biotin PEG_6 ligand in 1 mg of FNDs
$7.654 \times 10^{-9} \times N_A = 9.841 \times 10^{-9} \times 6.02 \times 10^{23} = \underline{4.608 \times 10^{15}\ \text{molecules}}$ of PEG biotin in 1 mg of FNDs.

Density of diamond: 3.51 g/mL= 3510 mg/cm^3 = 3.51×10^{-18} mg/nm^3
Volume of 80 nm sized FND: $4/3\pi R^3 = 4/3\pi(40)^3 = 268082.573$ nm^3
Volume x Density = weight of one FND = 9.409×10^{-13} mg/ea
1 mg/9.409×10^{-13} mg/ea = $\underline{1.062 \times 10^{12}}$ of FNDs in 1 mg

So, 4.608×10^{15} molecules of PEG biotin in 1mg of FNDs represents 4.608×10^{15} in 1.062×10^{12} FNDs

For 1 FND there is $4.608 \times 10^{15} / 1.062 \times 10^{12} = 4338$ pegylated (6) biotin per nanodiamond.

2. Quantifying biotin on nanodiamond surface.

To determine the amount of biotin on the ND surface, we used a fluorometric assay based on a fluorescent biotin construct (Biotin-4-Fluorescein) that quenches on binding to avidin. The assay relies on the fluorescent biotin construct to determine the concentration of free streptavidin remaining in solution after mixing a known a concentration of streptavidin with a known mass of biotinylated NDs (Fig. S3). A known amount of enriched biotinylated NDs (Enriched ND-Biotin), unenriched biotinylated NDs (Control ND-biotin), and unfunctionalized NDs (Control ND) were individually mixed with a proportional amount of 160 nM streptavidin in bead buffer (1 X PBS with 0.3% BSA and 0.01% TWEEN-20 (Sigma Aldrich #P9416)). The streptavidin solution was added to a separate vial without NDs as a control for streptavidin binding (Control Streptavidin). The diamonds were dispersed by sonication for ten minutes and then incubated on a rotator (1 turn/s) for one hour. The ND dispersions were then centrifuged (13,400 x *g* for 10 minutes) and the supernatants were collected. The streptavidin present in the supernatants was determined by mixing 20 µL of supernatant with 20 µL of a 40 nM solution of Biotin-4-Fluorescein (biotin-FITC) (Molecular probes, Thermo-Fisher Scientific #B10570), incubating for 15 minutes for the free streptavidin to bind the biotin-FITC, and measuring the fluorescence. The concentration of streptavidin present in the supernatant was calculated by comparing the measured fluorescence with a standard curve of fluorescence intensity of 20 nM biotin FITC as a function of streptavidin concentration. The amount of biotin present on the nanodiamonds was subsequently determined from the concentration of streptavidin in the supernatants.

As can be seen in Fig. S3, the fluorescence of a 1:1 mixture of the supernatant from the enriched ND-Biotin sample and 40 nM biotin-FITC resulted in a fluorescence of 212 RFU, which corresponds to a streptavidin concentration of ~ 0.9 nM in the mixture of biotin-FITC and supernatant. Thus, the streptavidin concentration in the supernatant alone of the Enriched ND-Biotin sample was 1.8 nM. Likewise, the streptavidin concentration in the supernatant of the control ND-Biotin sample was calculated to be 6.4 nM. The lower concentration of unbound streptavidin in the supernatant of the enriched ND-Biotin sample than the supernatant of the control ND-Biotin sample is consistent with the expected increase in surface-bound biotin on the enriched NDs.

The surface biotin density on the enriched and unenriched NDs coupled with amine-PEG2000-biotin was also determined by measuring the concentration at which the biotin was saturated by streptavidin[1] (Fig. 4). Aliquots of biotinylated nanodiamonds (~90 pM) in bead buffer were mixed with increasing concentrations (0-100 nM) of free streptavidin (Sigma Aldrich #S4762) and the hydrodynamic diameter of each sample was measured by dynamic light scattering (DLS) (Wyatt DynaPro NanoStar, cuvette based DLS) after a 30-minute incubation. Average particle size (intensity-weighted average hydrodynamic diameter) was measured by regularization methods with vendor supplied software. Because streptavidin is multivalent, the biotinylated nanodiamonds aggregate at sub-saturating concentrations of streptavidin. The biotinylated nanodiamonds will no longer aggregate when the biotins are saturated with streptavidin, at this concentration of streptavidin the average diameter decreases but remains slightly larger than the bare particles because of the bound streptavidin (*c.f.* Fig. 4 enriched ND data). The streptavidin concentration at which the hydrodynamic radius of the biotinylated nanodiamonds decreases provides an estimate of the concentration of biotin on the surface of the nanodiamonds.

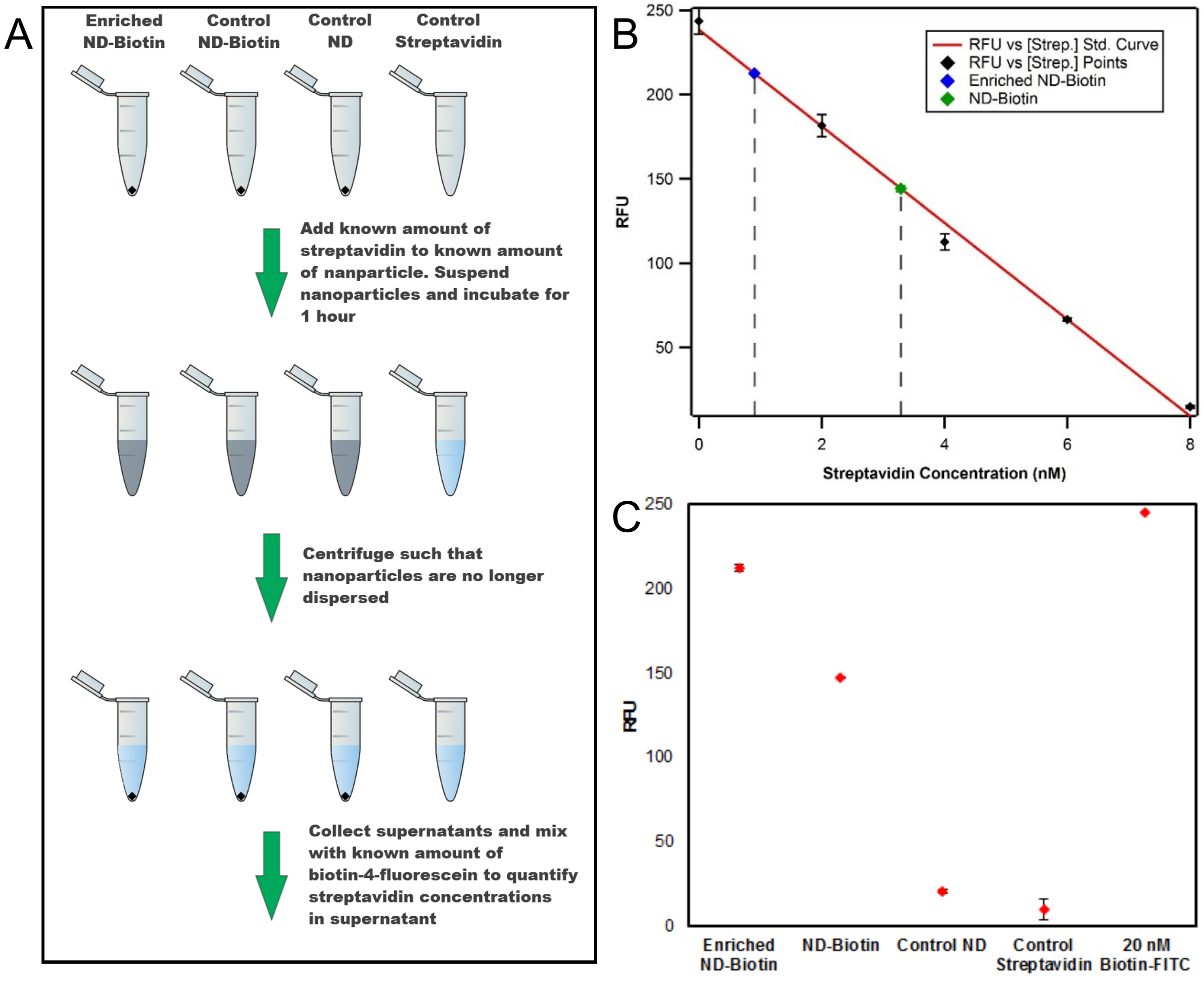

Figure S3: (A) Schematic of the assay used to determine the biotin content on the nanodiamond surfaces. (B) A standard curve of the measured fluorescence of 20 nM biotin-FITC as a function of streptavidin concentration. The measured fluorescence of a 1:1 mixture of 40 nM biotin-FITC and the supernatant from the Enriched ND-biotin and Control ND-Biotin samples and corresponding streptavidin concentrations of these two solutions is also shown. (C) The measured

fluorescence intensities of 1:1 mixtures of the various supernatants in (A) and 40 nM biotin-FITC and of 20 nM biotin-FITC as a reference for the maximum possible fluorescence.

3. Evanescent wave penetration depth measurement and calibration.

The detailed procedure for making 5 kilo-base-pair (kb) DNA templates, magnetic tweezers sample cells, and the combined magnetic tweezers-total internal reflection fluorescence instrument are described in detail elsewhere.[2] In brief, to measure the evanescent wave penetration depth (*d*), we first prepared a sample cell containing 5 kb DNA tethers, the two ends of which are attached to the surface and a streptavidin coated superparamagnetic bead (Invitrogen, Dynabeads MyOne, Streptavidin T1). For labeling magnetic beads with FND-biotin, 40 µl of 0.1 mg/ml FND-biotin was introduced into the sample cell to bind streptavidin on the magnetic beads. After 30 min incubation, unbound FND-biotin particles were washed out with 1ml of bead buffer. The penetration depth measurement was performed using a custom-built magnetic tweezers combined with total internal reflection fluorescence microscopy.[2] A rotationally constrained DNA molecule attached to a magnetic bead labelled with an FND was identified. All measurements were done at a force of 2 pN to reduce Brownian motion of the bead. The magnets were rotated in the counterclockwise direction to over-twist (positively supercoil) the DNA. Bright field images collected with a CMOS camera (JAI GO-2400-PMCL) at 200 Hz and fluorescent images collected with an EMCCD (Andor iXon+ DU-897) at 50 Hz were simultaneously acquired. DNA extension was measured by real-time tracking of the bead position from individual bright field images.[3] The fluorescent intensity of FND attached to the bead was estimated in a two-step procedure: First, the center of the FND fluorescence intensity distribution was obtained using an algorithm adapted from the Mosaic 2-D particle tracking code.[4] Second, the sum of the pixel intensities of the FND, within a cut-off radius (set at 90% of the total intensity), was calculated for each video frame. The fluorescent intensity calculated in this manner was plotted as function of the bead height and fitted with a single exponential to determine the evanescent wave penetration depth.

4. FND-biotin optical trapping.

FND-biotin was coated with streptavidin as follows: 0.2 mg FND-biotin was incubated with 1 µM streptavidin (SA) in 0.5 ml of bead buffer overnight at room temperature. To remove unbound SA, the mixture was centrifuged for 30 min at 13.4 kG and the supernatant was removed. The pellet was resuspended in 1 ml of bead buffer and vortexed to completely resuspend the FNDs. This wash step was repeated three times and the final pellet was resuspended in 200 µl of bead buffer. For tethering DNA with FND-biotin-SA, the streptavidin coated FND-biotin (FND-biotin-SA) was sonicated for 5 min and stored at 4° C until used. The procedures for making a 11 kilo-base DNA substrate, and a sample cell containing DNA tethers were previously described in detail.[5] To attach FND-biotin-SA to biotinylated end of DNA molecules, FND-biotin-SA was introduced into a sample cell containing surface tethered DNA, the free end of which contains a biotin. The sample cell was incubated at 4°C overnight and the unbound ND particles were removed by washing with 1 ml of bead buffer.

Assays were performed in a sample cell made of two coverslips (60 mm x 24 mm; #1 thickness; Corning Glass, Corning, NY) attached perpendicular to each other via a double-sided adhesive tape (volume = ~30 - 35 µL). To reduce nonspecific binding, the surface was coated through incubation with 1 mg/ml BSA in MilliQ water.

The optical trap is based on a published design.[6] It is built around an Olympus IX-71 inverted microscope with a UPlanApo/IR 60X magnification 1.20 numerical aperture water immersion objective (Olympus, Center Valley, PA) using a 5W 1064 nm wavelength laser (J20I-BL-106C; Spectra-Physics, Mountain View, CA) as the trapping laser source. Fluorescence images were acquired using an Andor iXon DV897EC-BV EMCCD camera (Andor – Oxford Instruments, South Windsor, CT) in combination with a TRITC long-pass widefield imaging filter cube (Olympus, Center Valley, PA). Typically, a laser power of ~75 mW was used to trap the particles at the specimen plane. Details of a similar optical trap are described in published articles.[7,8] Data was sampled at 20 kHz using a digital signal processing board (Innovative Integration, Cammarillo, CA) from a quadrant photodiode detector (Current Designs, Philadelphia, PA) that sampled the forward scattered laser light. Custom software written in C was used for data acquisition, and LabVIEW (National Instruments, Austin, TX) was used for further analysis.

FNDs tethered by DNA to the surface of the flow-cell were optically trapped, which imposes a three-dimensional restoring force on the trapped ND that can be characterized as a spring with stiffness k.[9] The position of the optically trapped nanodiamond was recorded via the forward scattered light collected on a quadrant photodiode and the power spectrum of the motion was computed. The power spectrum of an optically trapped object is described by a Lorentzian function that is characterized by an amplitude and a roll-off frequency, from which the trap stiffness can be obtained.[9]

5. Synthetic Methodology

5.1. General method for Carboxylic acid enrichment of oxidized FNDs

Detonation nanodiamonds (70 mg) (Adamas nanotechnologies) were placed in a glass vial along with a Teflon-coated stir bar and dried under vacuum by heating with a heat gun for ten minutes. The dried diamonds were allowed to cool under an argon environment. Upon cooling, 3 mL of a 2 M lithium aluminum hydride in THF solution (Sigma-Aldrich) was added to the vial slowly. The vial was sealed and sonicated to disperse the diamonds in the THF solution. The reduction reaction was kept at 65 °C for 48 h with vigorous stirring. After the reaction was complete, the dispersion was cooled in an ice bath and quenched slowly with drop-wise addition of 1 M HCl. The diamonds were then thoroughly washed with 1 M HCl, 1 M NaOH and finally DI water until the pH of the supernatant was slightly less than seven. The reduced diamonds were subsequently lyophilized into a powder form in order to obtain an IR spectrum. Upon obtaining an IR spectrum, 50 mg of the reduced diamonds and a Teflon-coated stir-bar were placed into a glass vial and again dried under vacuum using a heat gun for ten minutes. After heating, the dried diamonds were allowed to cool in an argon environment. The diamonds were then dispersed in 1.5 mL anhydrous benzene (Sigma-Aldrich) through sonication and stirring. To this suspension, 1.5 mg of rhodium (II) acetate dimer (Sigma-Aldrich) and 20 µL of *tert*-butyl diazoacetate (Sigma Aldrich) were added. The vial was flushed with argon, sealed, and kept at 80 °C for 48 h. The diamonds were subsequently isolated by centrifugation and washed thoroughly with dichloromethane (DCM) (Sigma-Aldrich) to remove any unreacted reagents. The diamonds were then allowed to dry for several hours under vacuum. After drying, 48 mg of the diamonds were transferred into a glass vial along with a Teflon-coated stir bar and 2 mL of 99% trifluoroacetic acid (Sigma-Aldrich) was added. The diamonds were dispersed in the trifluoroacetic acid by sonication. After 2 d at room temperature in the trifluoroacetic acid, the diamonds were again isolated by centrifugation and washed

thoroughly with water and methanol and dried under vacuum. A small amount of the diamonds was used to obtain an FTIR spectrum.

5.1.1. Coupling carboxylic acid enriched FNDs to amine-PEG$_6$-azide

To attach O-(2-aminoethyl)-O′-(2-azidoethyl) pentaethylene glycol (amine-PEG-azide) (Sigma-Aldrich) onto the carboxylic acid-enriched detonation nanodiamonds, enriched diamonds (20 mg) and a stir bar were dried under vacuum with a heat gun for 10 min. To this, 7 mL of thionyl chloride (97%, Sigma-Aldrich) was added. The vial was sealed, and the diamonds were sonicated to suspend them in the thionyl chloride. The dispersion was stirred and heated to 70 °C for 48 h. The diamond dispersion was allowed to cool to room temperature and then centrifuged (3270 x g for 1 hour). The thionyl chloride supernatant was removed and the pellet of acyl chloride functionalized nanodiamonds was kept under vacuum for four hours to remove residual thionyl chloride. Air exposure was minimized to prevent the reactive acyl chlorides from coming into contact with moisture. Dry acyl chloride nanodiamonds (16 mg) were suspended in 1 mL of DCM (Sigma-Aldrich). To this, 100 µL of O-(2-aminoethyl)-O′-(2-azidoethyl) pentaethylene glycol (amine-PEG-azide) and 50 mg 4-dimethylaminopyridine (DMAP) was added. The diamond dispersion was kept at 39 °C for 1 d. The diamonds were then isolated by centrifugation and thoroughly washed with DCM and methanol to remove unreacted reagents. The diamonds were dried under vacuum and a few milligrams of the dried powder were used to obtain an infrared spectrum.

5.1.2. Coupling carboxylic acid enriched FNDs to amine-PEG$_{2000}$-biotin

Amine-PEG-biotin (MW: 5000, Nanocs) was attached onto fluorescent nanodiamonds (Adamas Nanotechnologies, acid treated) in an identical manner by activating the carboxylic acids with thionyl chloride and subsequently suspending the diamonds in DCM and adding DMAP and the amine-PEG-biotin. The fluorescent nanodiamonds were separated into two batches, one that was enriched for carboxylic acids following the procedure outlined above (ND-COOH-Enriched), and another that was used as is (ND-COOH). Biotin was attached to both batches of diamonds using identical procedures and identical amounts of diamond, dichloromethane, DMAP, and amine-PEG-biotin (Fig. S4).

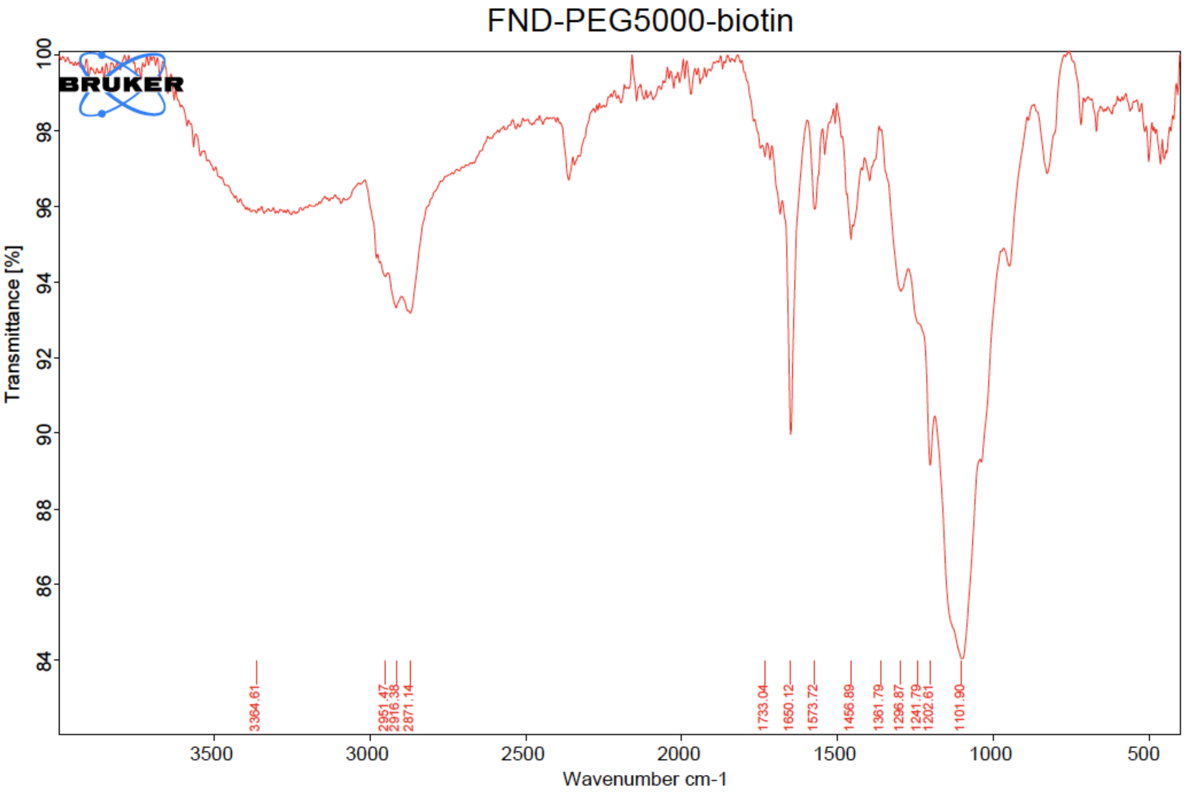

<u>Figure S4:</u> FTIR spectrum of FND-amine-PEG2000-biotin.

5.2. <u>General method for Carboxylic acid enrichment of reduced FNDs</u>

Reduced fluorescent nanodiamond powder (80 nm diameter) was supplied by Adámas Nanotechnologies, Inc., USA. (Elemental Analysis C : 96.13 % H < 0.10 % N : <0.10 %)
The nanodiamonds, produced by high pressure high temperature (HPHT) static synthesis, were reduced with 2 M lithium aluminum hydride in THF solution for 48h at 65 °C. The dispersion was cooled in an ice bath and quenched slowly with drop-wise addition of 1 M HCl. The diamonds were then thoroughly washed with 1 M HCl, 1 M NaOH and finally DI water until neutral pH. The reduced and purified diamonds were subsequently lyophilized into a powder form and used as a starting material for subsequent functionalization.

5.2.1. <u>Carboxylic acid enrichment of FNDs</u>

Fluorescent nanodiamonds (70mg) were dried under vacuum by heating with a heat gun for 10 minutes and cooled under an argon environment. Nanodiamonds were dispersed with sonication in anhydrous benzene (1.5 mL, Sigma-Aldrich) with Rhodium (II) acetate dimer (1.5 mg, Sigma-Aldrich) and *tert*-butyl diazoacetate (20 µL, Sigma-Aldrich). The vial was flushed with argon, sealed, and kept at 80 °C for 48 h. The nanodiamonds were subsequently isolated by centrifugation and washed thoroughly with DCM to remove any unreacted reagents and dried for several hours under vacuum. Trifluoroacetic acid (2 mL) was added the dried nanodiamonds and dispersed by sonication. After 48 h at room temperature, the nanodiamonds were isolated by centrifugation and

washed thoroughly with water and methanol and dried under vacuum. A small amount of the diamonds was used to obtain an FTIR spectrum.

5.2.2. Synthesis of acyl chloride FNDs

Carboxylic acid enriched FNDs (30 mg) were dried under vacuum with a heat gun for ten minutes. Neat thionyl chloride (5 mL) was added to the sealed vial and sonicated to suspend the NDs. The dispersion was stirred and heated at 70 °C for 48 h. The thionyl chloride supernatant was removed after cooling the vial to room temperature and centrifugation (3270 x g for 1 h). The pellet of acyl chloride functionalized nanodiamonds was kept under vacuum for 4 hours to remove residual thionyl chloride (Fig. S5).

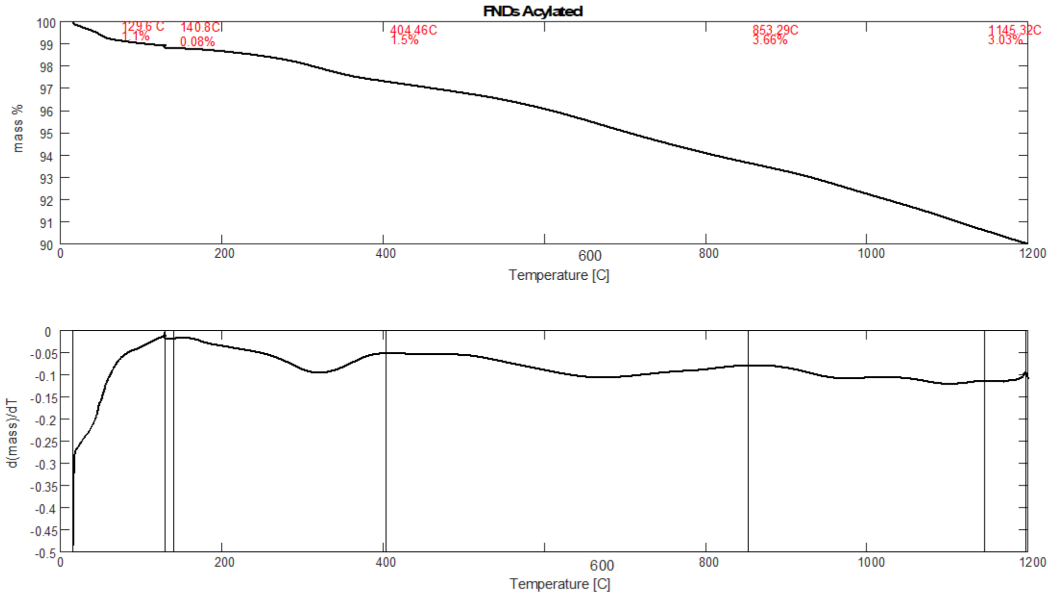

Figure S5: TGA (top) and DGT (bottom) curves for acyl chloride FNDs.

5.2.3. Coupling of carboxylic acid enriched FNDs with tags

a) ND-amido-PEG$_6$-azide Dry chloroacetylated fluorescent nanodiamonds (25 mg) were suspended in dry DCM (2 mL) with O-(2-aminoethyl)-O′-(2-azidoethyl) pentaethylene glycol (Broadpharm) (azido-PEG$_6$-amine, 100 µL), DMAP (50 mg) and triethylamine (100 µL). The nanodiamond suspension was kept at 39 °C for 24 h. The functionalized nanodiamonds were isolated by centrifugation, thoroughly washed with DCM and methanol to remove unreacted reagents, and dried under vacuum. A few milligrams of the dried powder were used to obtain an infrared spectrum and TGA (Fig. S6).

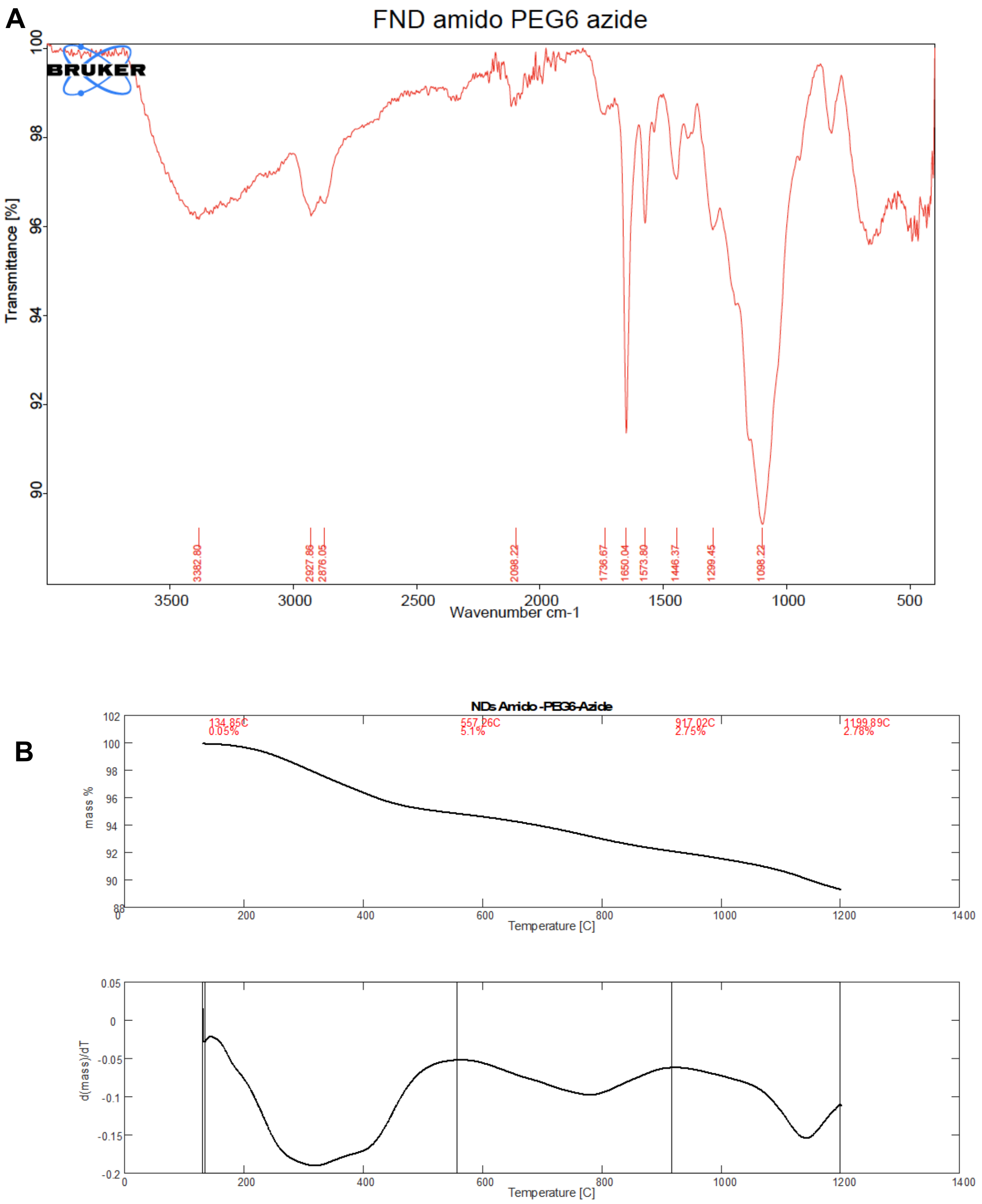

Figure S6: (A) FTIR spectrum and (B) TGA (top) and DGT (bottom) curves for FND-amido-PEG_6-azide.

b) ND-amido-PEG$_6$-Propargyl Dry chloroacetylated fluorescent nanodiamonds (25 mg) were suspended in dry DCM (2 mL) with propargyl-PEG$_6$- Amine (100 µL, Broadpharm), DMAP (50 mg) and triethylamine (100 µL). The nanodiamond suspension was kept at 39 °C for 24h. The functionalized nanodiamonds were isolated by centrifugation, thoroughly washed with DCM and methanol to remove unreacted reagents and dried under vacuum. A few milligrams of the dried powder were used to obtain an infrared spectrum and TGA (Fig. S7). Elemental Analysis indicated C: 92.21% H: 0.57% N: 0.23%. The expected elemental analysis for amido-PRG$_6$-Propargyl alone is C: 56.28%; H: 8.70%; N: 3.45%; O: 31.56%. Since the nanodiamonds contribute a large background carbon signal but low hydrogen and nitrogen signals (< 0.1%, see above), we confirm the identity of the surface functional groups by comparing the expected and measured ratio of nitrogen to hydrogen. For amido-PRG$_6$-Propargyl functionalized nanodiamonds the measured N/H ratio was 0.40, in excellent agreement with the expected N/H ratio of 0.39.

A

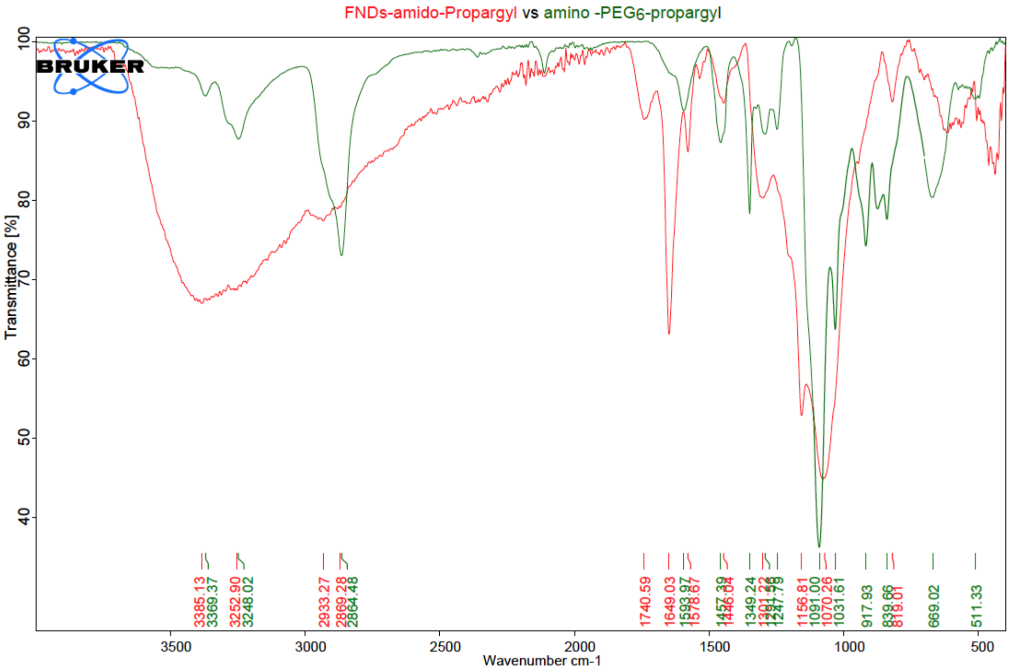

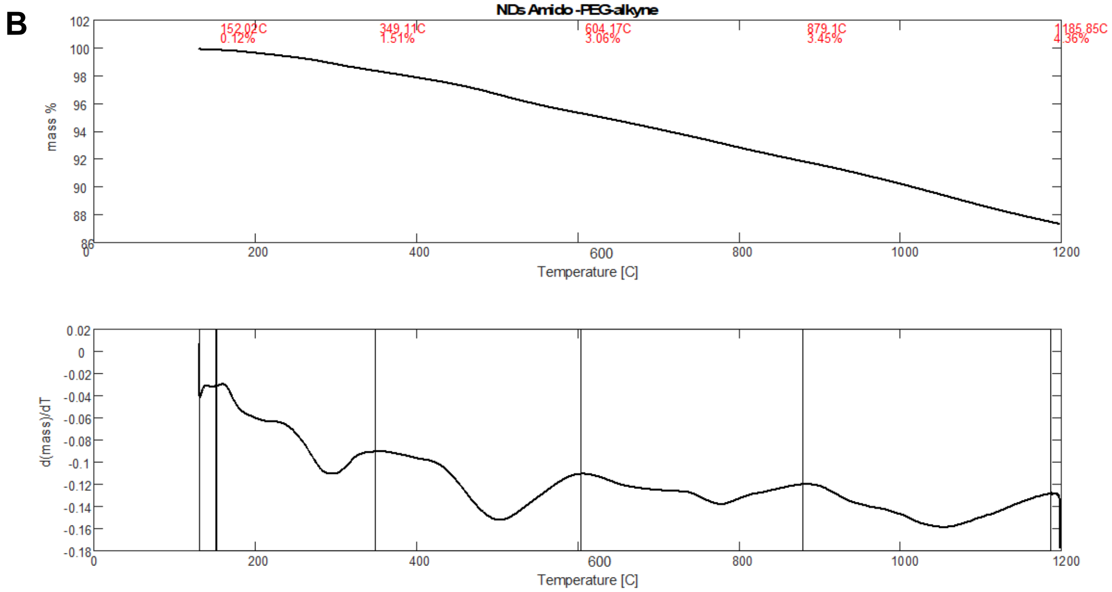

Figure S7: (A) FTIR spectrum and (B) TGA (top) and DGT (bottom) curves for FND-amido-PEG$_6$-Propargyl.

c) ND-amido-PEG$_6$-Biotin Dry chloroacetylated fluorescent nanodiamonds (25 mg) were suspended in dry DCM (2 mL) with biotin-PEG$_6$-amine (100 µL, Broadpharm), DMAP (50 mg) and triethylamine (100 µL). The nanodiamond suspension was kept at 39 °C for 24 h. The functionalized nanodiamonds were isolated by centrifugation, thoroughly washed with DCM and methanol to remove unreacted reagents and dried under vacuum. A few milligrams of the dried powder were used to obtain an infrared spectrum and TGA (Fig. S8). Elemental Analysis indicated: C: 91.38%; H: 0.48%; N: 0.31%, giving a measured (N/H) ratio of 0.88. The expected elemental analysis for amido-PEG6-Biotin is: C: 52.07%; H: 8.09%; N: 9.00%; O: 25.69%; S: 5.15%. The resulting expected (N/H) ratio of 1.11 is in good agreement with the measured (N/H) ratio of 0.88.

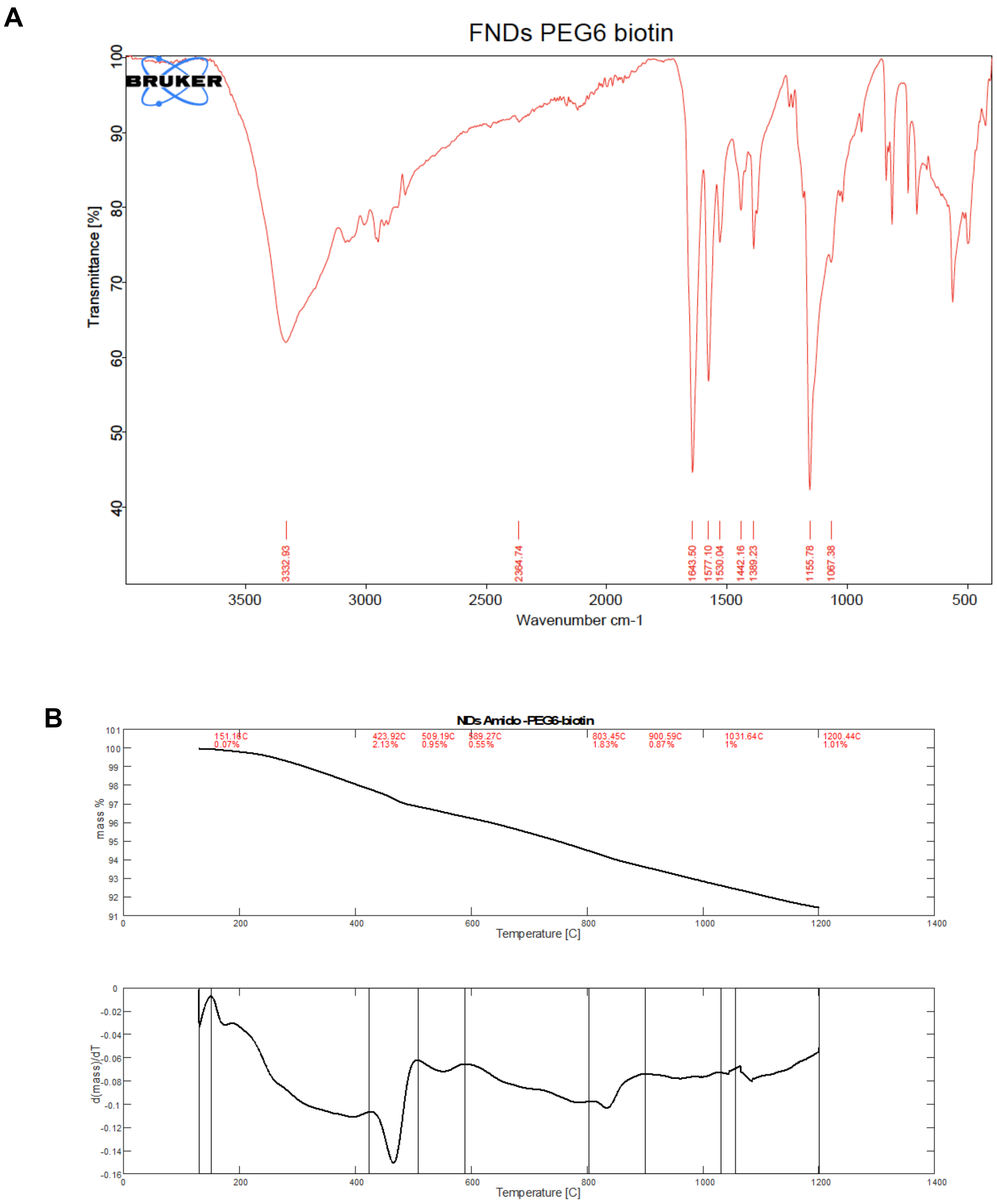

Figure S8: (A) FTIR spectrum and (B) TGA (top) and DGT (bottom) curves for FND-amido-PEG6-Biotin.

d) ND-amido-ethyl-PEG$_4$-Maleimide Dry chloroacetylated fluorescent nanodiamonds (25 mg) were suspended in dry DCM (2 mL) with maleimide ethyl-PEG$_4$-amine TFA salt (100 µL, Abosyn), DMAP (50 mg) and triethylamine (100 µL). The nanodiamond suspension

was kept at 39 °C for 24 h. The functionalized nanodiamonds were isolated by centrifugation, thoroughly washed with DCM and methanol to remove unreacted reagents and dried under vacuum. A few milligrams of the dried powder were used to obtain an infrared spectrum and TGA (Fig. S9). Elemental Analysis indicated: C : 85.96 % H : 0.99 % N : 0.98 %, giving a measured (N/H) ratio of 0.98. The expected elemental analysis for amido-ethyl-PEG4-Maleimide is: C: 52.57%; H: 7.27%; N: 7.21%; O: 32.95%. The resulting expected (N/H) ratio of 0.98 is in excellent agreement with the measured (N/H) ratio of 0.98.

A

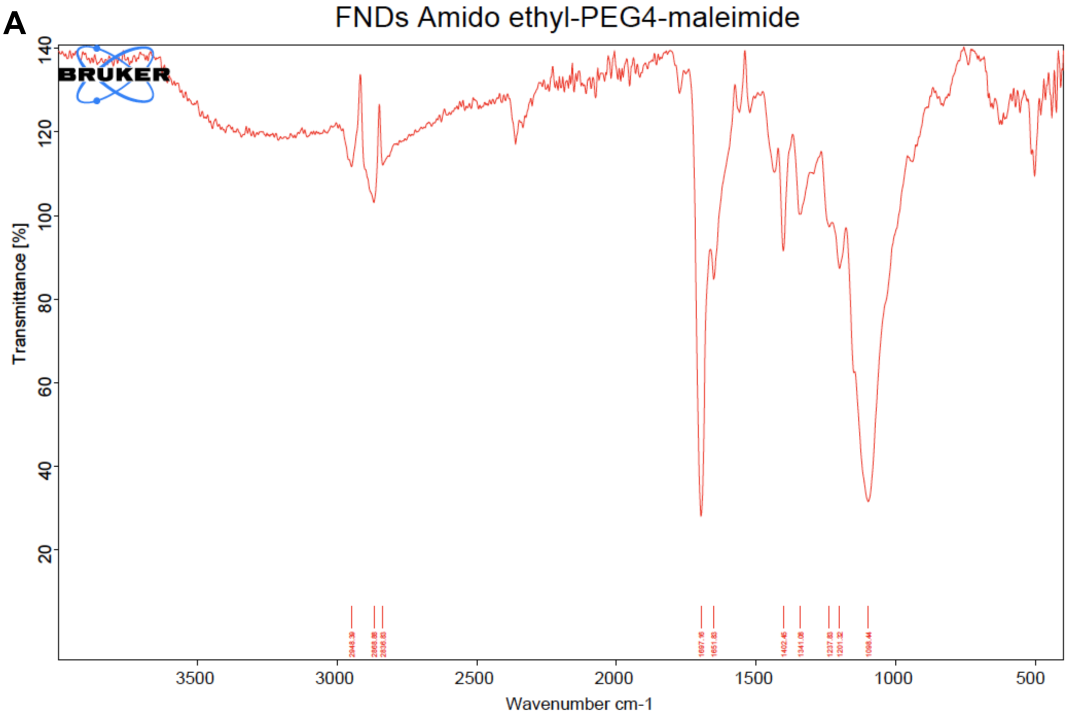

B

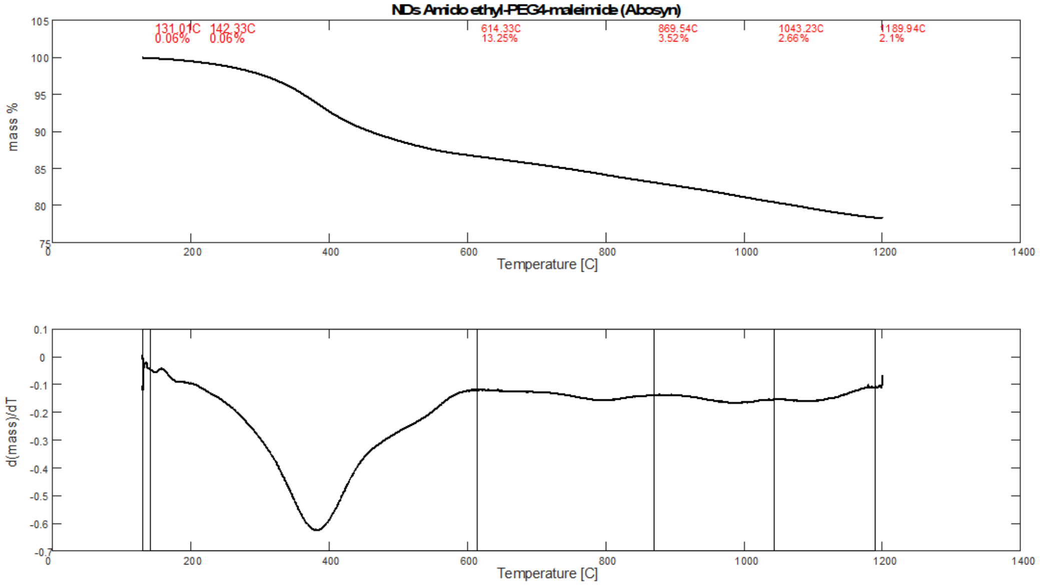

Figure S9: (A) FTIR spectrum and (B) TGA (top) and DGT (bottom) curves for FND-amido-ethyl-PEG4-Maleimide.

e) **ND-amido-PEG$_3$-amido-t-Boc-N** Dry chloroacetylated fluorescent nanodiamonds (25 mg) were suspended in dry DCM (2 mL) with t-Boc-N-amido-PEG$_3$-amine (100 µL, Broadpharm), DMAP (50 mg) and triethylamine (100 µL). The nanodiamond suspension was kept at 39 °C for 24h. The functionalized nanodiamonds were isolated by centrifugation, thoroughly washed with DCM and methanol to remove unreacted reagents and dried under vacuum. A few milligrams of the dried powder were used to obtain an infrared spectrum and TGA (Fig. S10).

A

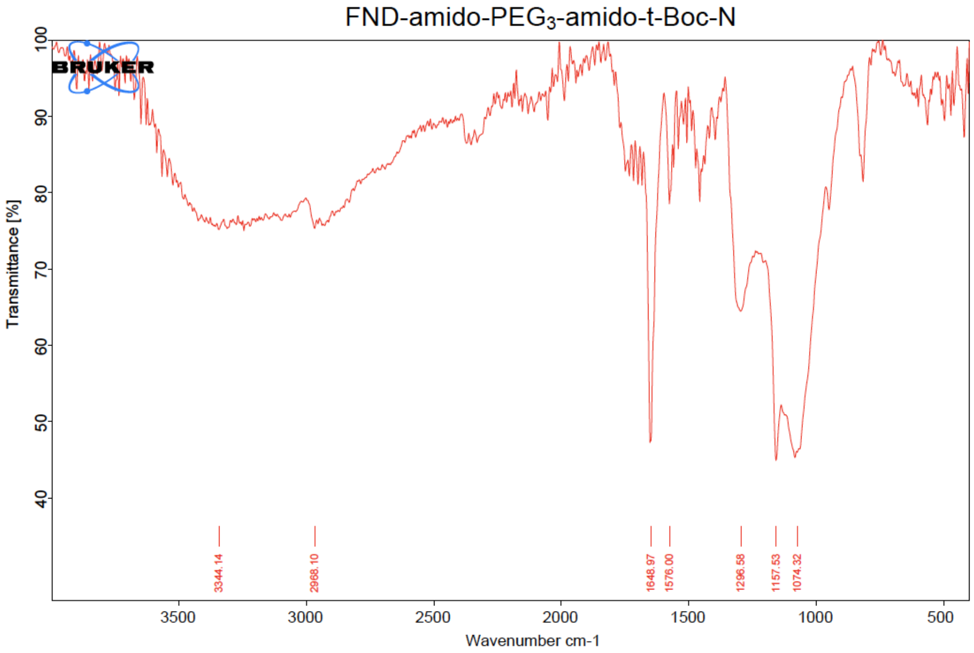

B

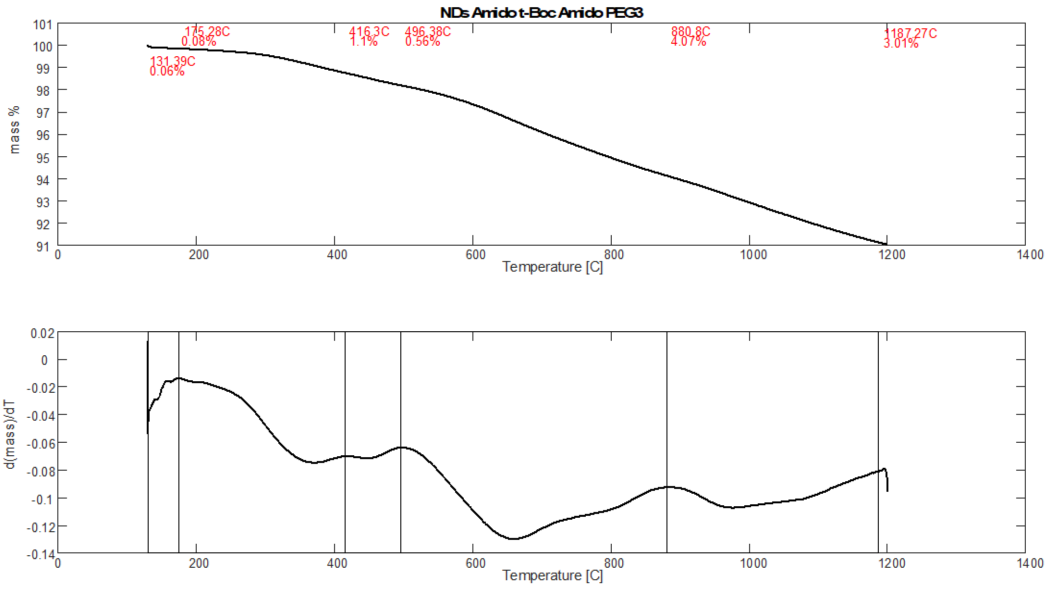

Figure S10: (A) FTIR spectrum and (B) TGA (top) and DGT (bottom) curves for FND-amido-PEG3-amido-t-Boc-N.

f) ND-amidoethyl-PEG$_4$-carboxilic acid Dry chloroacetylated fluorescent nanodiamonds (25 mg) were suspended in dry DCM (2 mL) with Amino-PEG$_4$-carboxylic acid (100 µL, Broadpharm), DMAP (50 mg) and triethylamine (100 µL). The nanodiamond suspension was kept at 39 °C for 24h. The functionalized nanodiamonds were isolated by centrifugation, washed with DCM and methanol to remove unreacted reagents and dried under vacuum. A few milligrams of the dried powder were used to obtain an infrared spectrum and TGA (Fig. S11).

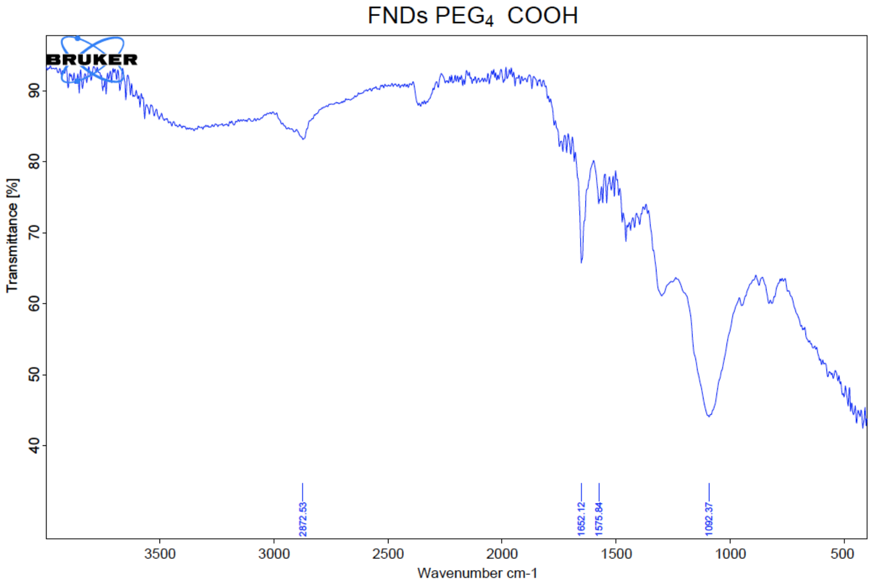

Figure S11: FTIR spectrum of FND-amidoethyl-PEG4-carboxilic acid.

g) ND-amidoethyl-PEG$_4$-acetyl chloride Neat thionyl chloride (5 mL) was added to a sealed vial containing prepared FND-amidoethyl-PEG$_4$-carboxilic acid particles and sonicated to suspend the FNDs. The dispersion was stirred and heated at 70 °C for 48 hours. After cooling, thionyl chloride supernatant was removed after centrifugation (3270 x g for 1 hour). The pellet of acyl chloride functionalized nanodiamonds was kept under vacuum for 4 hours to remove residual thionyl chloride. A few milligrams of the dried powder were used to obtain an infrared spectrum and TGA (Fig. S12). Elemental Analysis indicated C: 81.70%; H: 0.73%; N: 0.17%, giving a measured (N/H) ratio of 0.23. The expected elemental analysis amidoethyl-PEG4-acetyl chloride: C: 47.26%; H: 7.37%; Cl: 9.96%; N: 3.94%; O: 31.48%. The resulting expected (N/H) ratio of 0.53 is in reasonable agreement with the measured (N/H) ratio of 0.23.

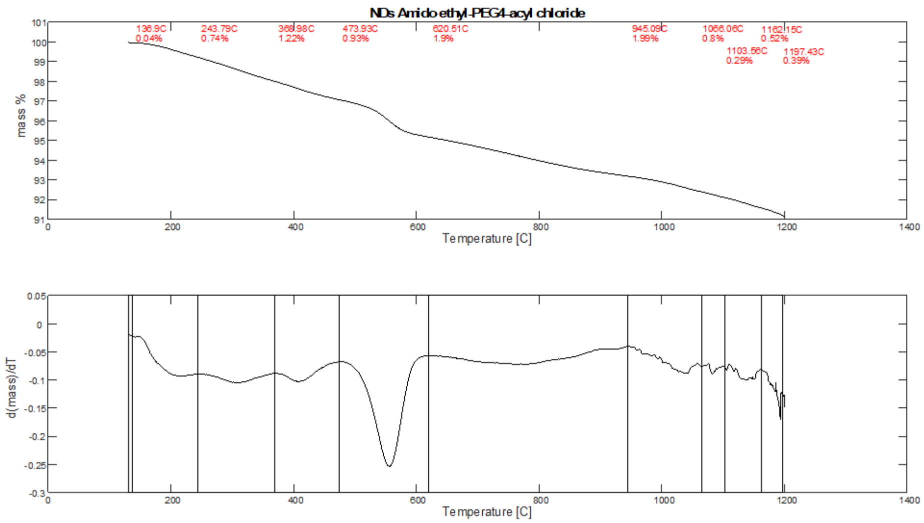

<u>Figure S12:</u> TGA (top) and DGT (bottom) curves for FND-amidoethyl-PEG4-acetyl chloride.

h) ND-amido-PEG$_4$-DBCO Dry chloroacetylated fluorescent nanodiamonds (25 mg) were suspended in dry DCM (2 mL) with DBCO-PEG$_4$-Amine (100 µL, Broadpharm), DMAP (50 mg) and triethylamine (100 µL). The nanodiamond suspension was kept at 39 °C for 24h. The functionalized nanodiamonds were isolated by centrifugation, thoroughly washed with DCM and methanol to remove unreacted reagents and dried under vacuum. A few milligrams of the dried powder were used to obtain an infrared spectrum and TGA (Fig. S13). Elemental analysis indicated C: 86.13%; H: 0.38%; N: 0.31%, giving a measured (N/H) ratio of 0.815. The expected elemental composition for amido-PEG$_4$-DBCO is: C: 64.01%; H: 6.76%; N 7.22%; O: 22%. The resulting expected (N/H) ratio of 1.01 is in excellent agreement with the measured (N/H) ratio of 0.815.

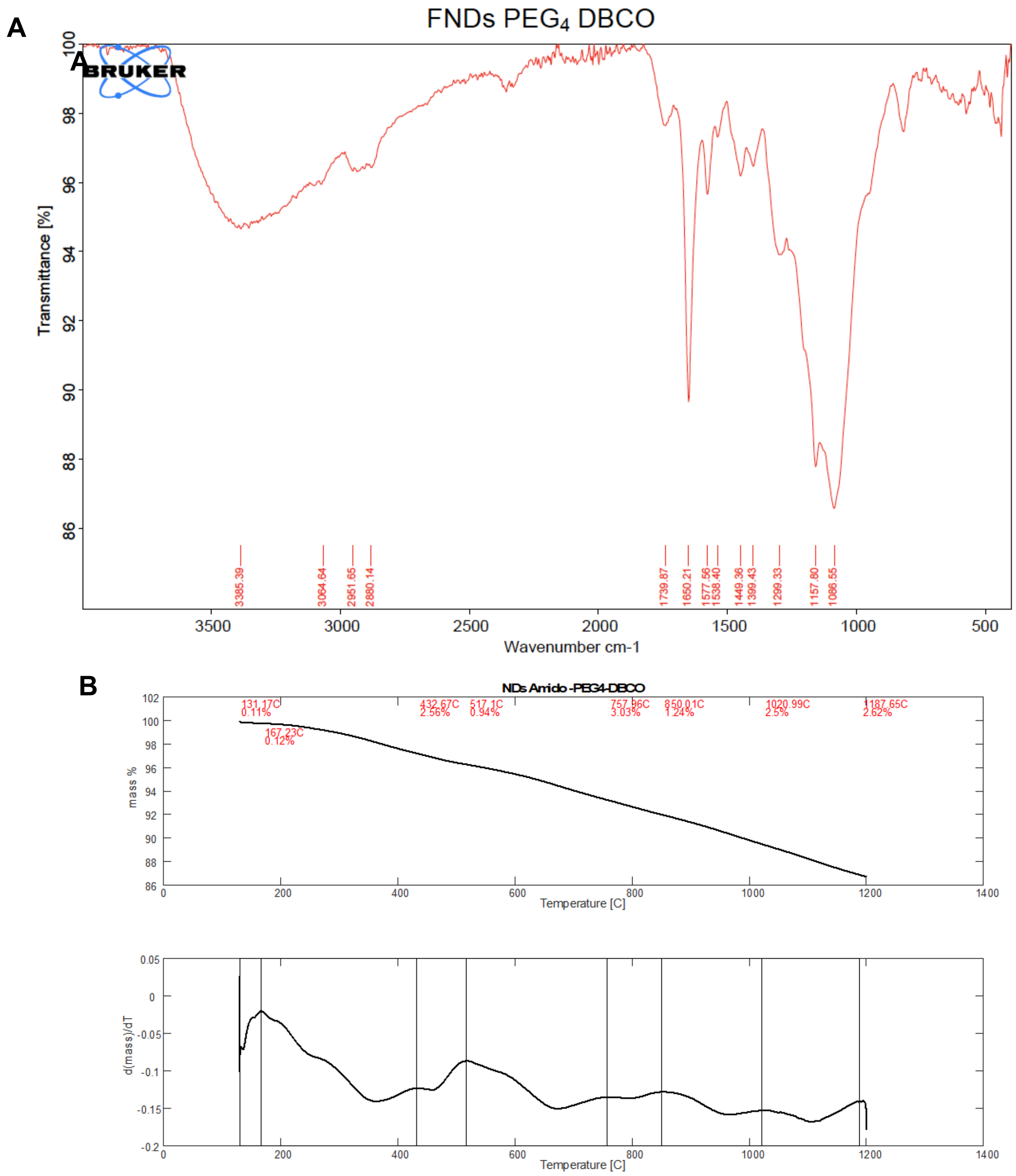

<u>Figure S13:</u> (A) FTIR spectrum and (B) TGA (top) and DGT (bottom) curves for FND-amido-PEG4-DBCO.

i) **ND-amido-Halo Tag** Dry chloroacetylated fluorescent nanodiamonds (25 mg) were suspended in dry DCM (2 mL) with HaloTag (100 µL, Amine-halotag, Ambeed), DMAP (50 mg) and triethylamine (100 µL). The nanodiamond suspension was kept at 39 °C for 24h. The functionalized nanodiamonds were isolated by centrifugation, thoroughly washed with DCM and methanol to remove unreacted reagents and dried under vacuum.

A few milligrams of the dried powder were used to obtain an infrared spectrum and TGA (Fig S14). Elemental Analysis indicated: C: 89.02%; H: 0.45% N: 0.17%, giving a measured (N/H) ratio of 0.38. The expected elemental composition for amido-Halo tag is: C: 52.79%; H: 8.86%; Cl: 11.98%; N: 4.74%; O, 21.63%. The resulting expected (N/H) ratio of 0.55 is in reasonable agreement with the measured (N/H) ratio of 0.38.

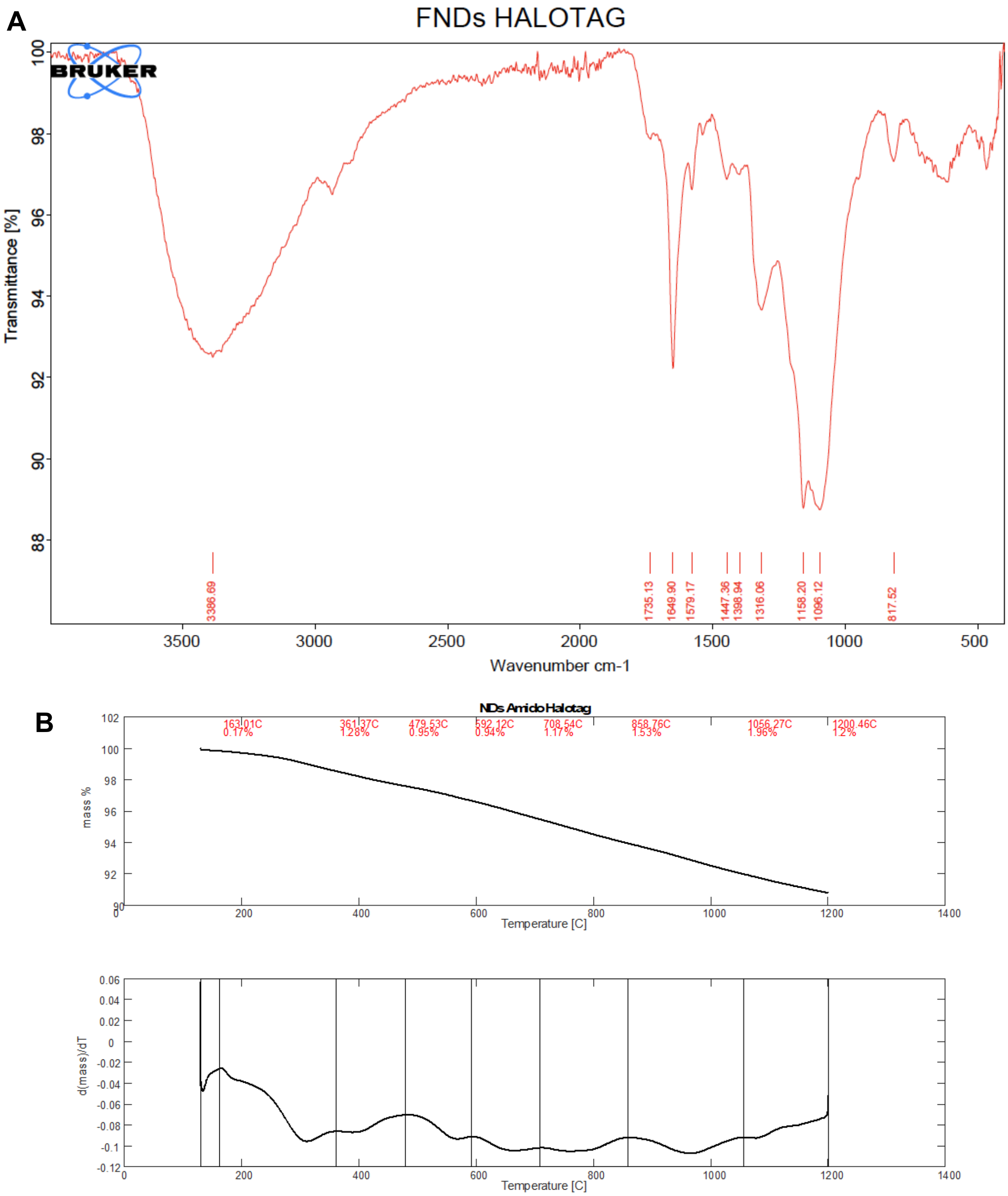

<u>Figure S14:</u> (A) FTIR spectrum and (B) TGA (top) and DGT (bottom) curves for FND-amido-Halo Tag.

j) ND-amido-SNAP Tag Dry chloroacetylated fluorescent nanodiamonds (25 mg) were suspended in dry DCM (2 mL) with 6-((4-(Aminomethyl)benzyl)oxy)-7H-purin-2-amine (100 µL, Amine-Snaptag, Click Chemistry), DMAP (50 mg) and triethylamine (100 µL). The nanodiamond suspension was kept at 39 °C for 24h. The functionalized nanodiamonds were isolated by centrifugation, thoroughly washed with DCM and methanol to remove unreacted reagents and dried under vacuum. A few milligrams of the dried powder was used to obtain an infrared spectrum and TGA (Fig. S15).

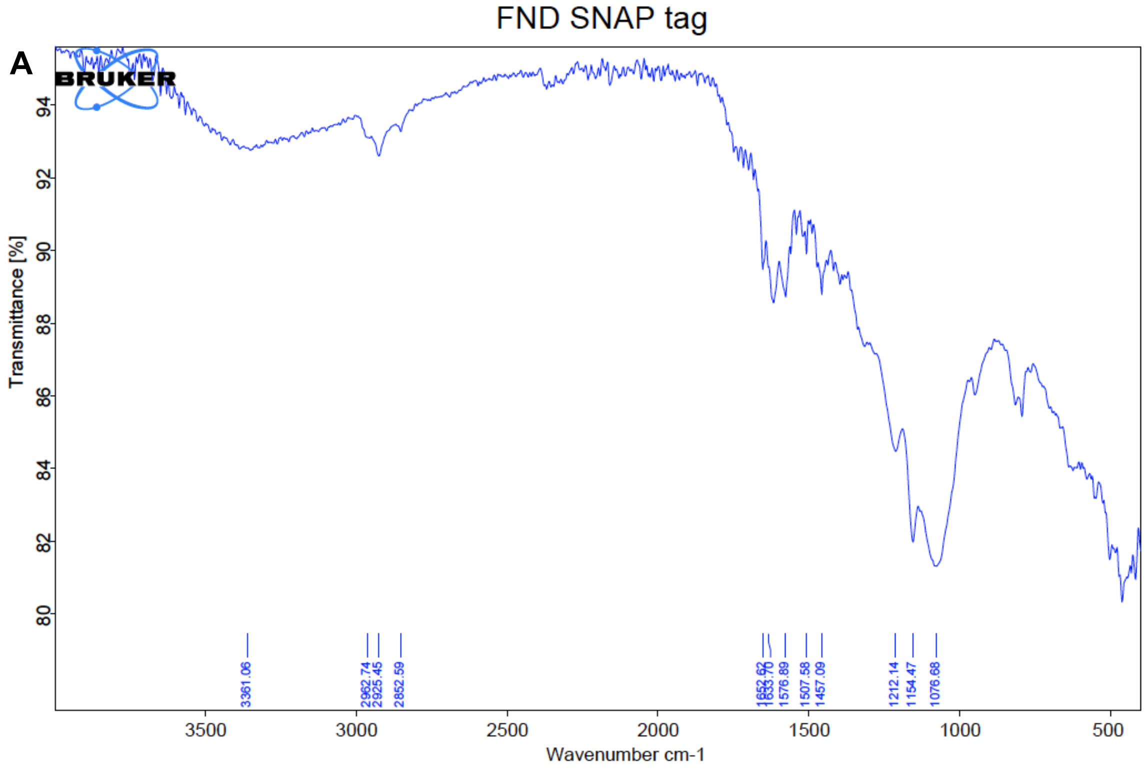

B

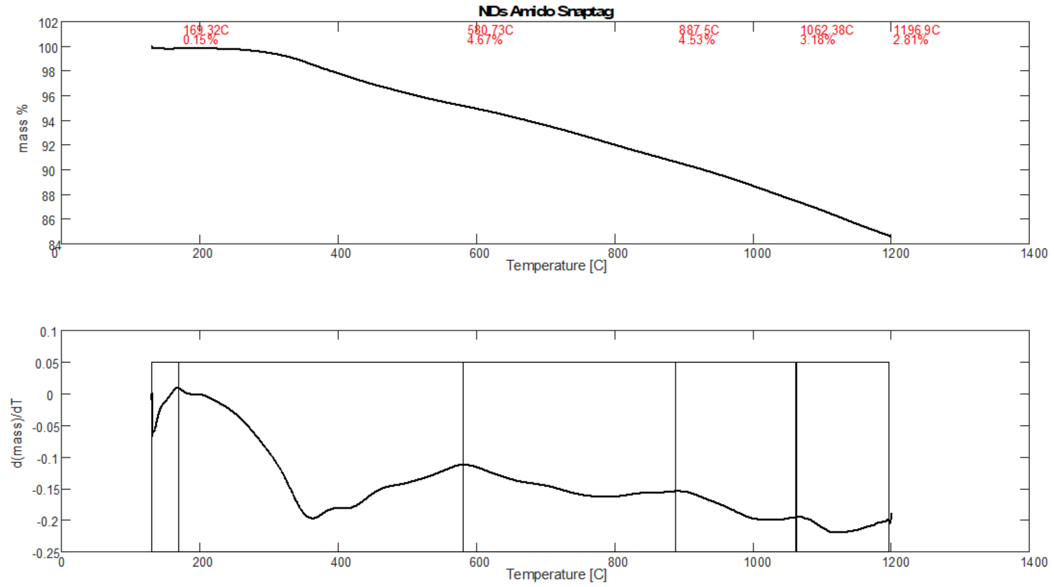

Figure S15: (A) FTIR spectrum and (B) TGA (top) and DGT (bottom) curves for FND-amido-SNAP Tag.

k) ND-amido-Clip Tag Dry chloroacetylated fluorescent nanodiamonds (25 mg) were suspended in dry DCM (2 mL) with 2-((4-(Aminomethyl)benzyl)oxy)pyrimidin-4-amine (100 µL, Amine-cliptag, Ambeed), DMAP (50 mg) and triethylamine (100 µL). The nanodiamond suspension was kept at 39 °C for 24h. The functionalized nanodiamonds were isolated by centrifugation, thoroughly washed with DCM and methanol to remove unreacted reagents and dried under vacuum. A few milligrams of the dried powder were used to obtain an infrared spectrum and TGA (Fig. S16). Elemental Analysis indicated: C: 90.58%; H: 0.49% N: 0.79%, giving a measured (N/H) ratio of 1.61. The expected elemental composition for amido-Clip Tag $C_{14}H_{16}N_4O_3$ is: C: 58.32%; H: 5.59%; N: 19.43%; O: 16.65%. The resulting expected (N/H) ratio of 3.47 is in reasonable agreement with the measured (N/H) ratio of 1.61.

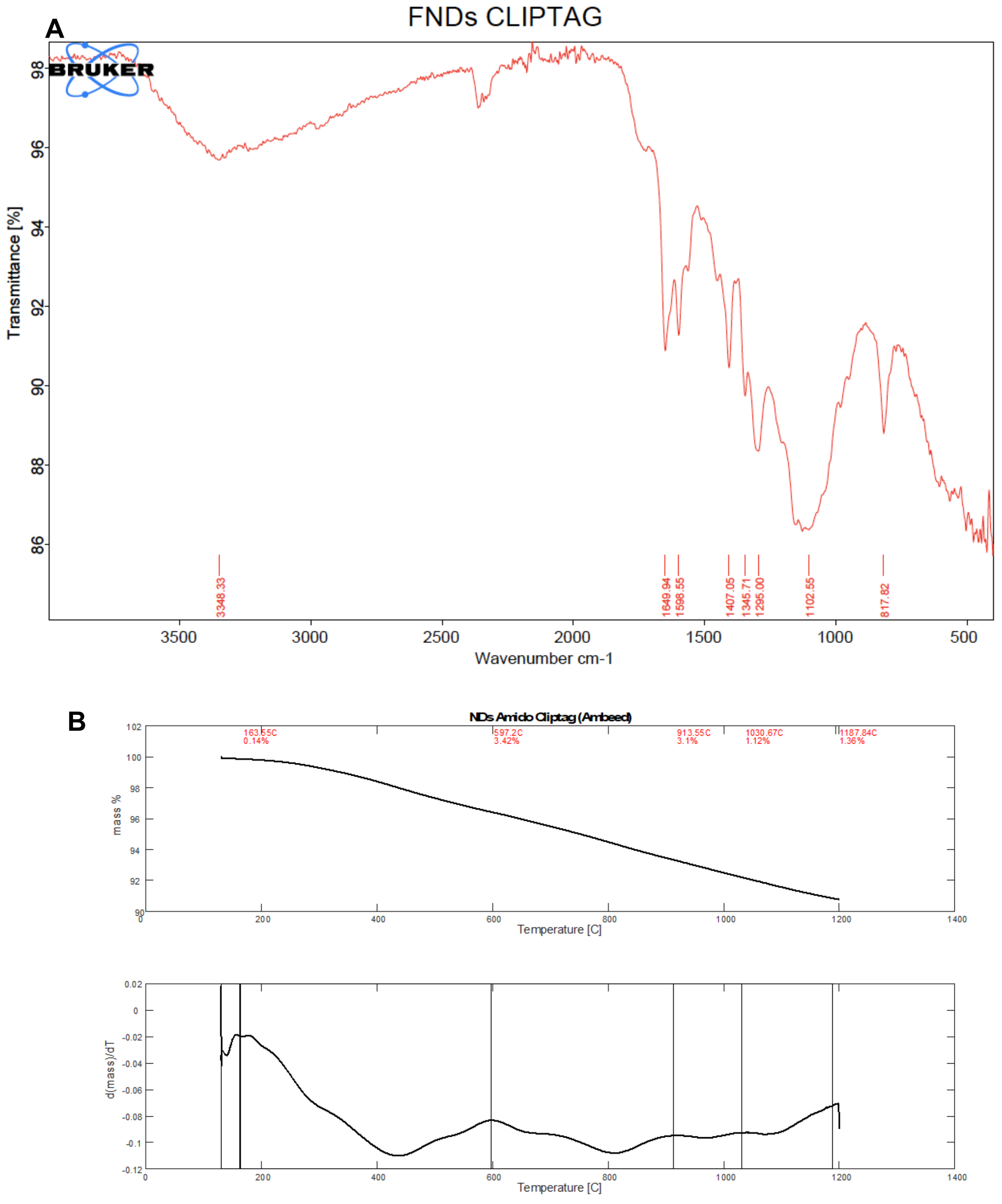

Figure S16: (A) FTIR spectrum and (B) TGA (top) and DGT (bottom) curves for FND-amido-Clip Tag.

REFERENCES

[1] H.-S. Jung, K.-J. Cho, Y. Seol, Y. Takagi, A. Dittmore, P. A. Roche, K. C. Neuman, *Adv. Funct. Mater.* **2018**, *28*, 1801252.

[2] Y. Seol, K. C. Neuman, in *Methods Mol. Biol.*, **2018**.

[3] T. Lionnet, J. F. Allemand, A. Revyakin, T. R. Strick, O. A. Saleh, D. Bensimon, V. Croquette, *Cold Spring Harb. Protoc.* **2012**, DOI 10.1101/pdb.prot067496.

[4] I. F. Sbalzarini, P. Koumoutsakos, *J. Struct. Biol.* **2005**, DOI 10.1016/j.jsb.2005.06.002.

[5] Y. Seol, A. C. Gentry, N. Osheroff, K. C. Neuman, *J. Biol. Chem.* **2013**, DOI 10.1074/jbc.M112.444745.

[6] K. Visscher, S. P. Gross, S. M. Block, *IEEE J. Sel. Top. Quantum Electron.* **1996**, DOI 10.1109/2944.577338.

[7] F. Vanzi, Y. Takagi, H. Shuman, B. S. Cooperman, Y. E. Goldman, *Biophys. J.* **2005**, DOI 10.1529/biophysj.104.056283.

[8] Y. Takagi, E. E. Homsher, Y. E. Goldman, H. Shuman, *Biophys. J.* **2006**, DOI 10.1529/biophysj.105.068429.

[9] K. C. Neuman, S. M. Block, *Rev. Sci. Instrum.* **2004**, *75*, 2787–2809.
